# Hand Book of Compound Medicines

**Published:** 1872-12

**Authors:** 


*Hand-Book of Compound Medicines; or the Prescriber's and Dispenser's Vade-Mecum.* By ARNOLD J. COOLEY. Philadelphia: J. B. Lippincott & Co., 1873; Buffalo: H. H. Otis. pp. 219.

This work, which is a curious combination of hospital and private prescriptions and patent medicine formulæ, is divided into two parts—Part I. containing formulas for pills, boluses, globules, grains and granules; and Part II. for mixtures. The work is of some value as containing, in some instances, valuable formulas; but it is not up to the times, many new and valuable medicines being entirely omitted. To one curious as to how medicines are compounded and what quack medicines are made of, it may be of sufficient value to warrant its perusal.

